# Resident doctor workforce wellbeing worldwide: Lessons between the UK and Australia

**DOI:** 10.1016/j.clinme.2026.100614

**Published:** 2026-07-02

**Authors:** Neeraj B. Bhala, Emily C. Taylor, Andrew F. Goddard, Ajay M. Verma

**Affiliations:** a Department of Gastroenterology, Royal Melbourne Hospital and University of Melbourne, Parkville, Victoria, Australia; b Derby Royal Infirmary, University Hospitals Derby and Burton NHS Foundation Trust, Derbyshire, UK; c Kettering General Hospital, University Hospitals Kettering and Northampton NHS Foundation Trust, Northamptonshire, UK

**Keywords:** UK, Australia, Healthcare, Workforce, Wellbeing

## Abstract

Healthcare systems fundamentally depend on the wellbeing and sustainability of their workforce. Physician burnout, workload strain and declining professional fulfilment are widely documented across high-income health systems. The UK and Australia share similar professional cultures and training pathways, but operate within distinct health system structures. Comparing these systems provides insight into the structural determinants of resident doctor wellbeing. Both countries face common challenges, including rising demand for care, domestic workforce shortages, and changing expectations of work–life balance. Data from both countries demonstrate persistently high levels of moral distress and intention to leave practice, alongside structural pressures including industrial action, training bottlenecks and changing workforce composition. Evidence on suicide risk further underscores the seriousness of workforce wellbeing as a public health issue. These shared outcomes highlight that structural differences do not protect resident doctors from distress. System-level reform and cultural change are essential to safeguard workforce wellbeing and health system sustainability.

## Introduction

Healthcare systems rely on the wellbeing, motivation and sustainability of their workforce. Over the past decade, physician burnout (increasingly (and more accurately) referred to as moral distress) has emerged as a major concern across health systems. This was intensified by the COVID-19 pandemic, but reflects longstanding structural pressures including workforce shortages, rising service demand and administrative burden.

The United Kingdom (UK) and Australia share similar medical training traditions and strong professional mobility between their workforces. However, their health systems differ substantially in structure, funding and workforce capacity. These differences provide an opportunity to consider how health system design may influence workforce wellbeing, especially among resident doctors (Table 1).

**Table 1:** Comparison of selected workforce indicators: UK vs Australia.

Indicator	United Kingdom	Australia	OECD average
Doctors per 1,000 population	∼3.4	∼4.2	∼3.9
Health expenditure (% GDP)	∼10.2%	∼10.3%	∼9.3%
Health expenditure per capita (USD PPP)	∼$5,500	∼$7,400	∼$4,986
International medical graduates (% of doctors)	∼28–30%	∼33–35%	∼18%
System structure	Predominantly publicly funded NHS	Mixed public–private system	Mixed models
Key workforce pressure	Staffing shortages and workload intensity	Workforce distribution and rural shortages	Varies by country/region

## The scale of the problem

Wellbeing surveys of doctors in both Australia and the UK present a mixed picture. While overall satisfaction with training remains high, significant levels of burnout, workload strain and declining professional fulfilment are evident.^1^, ^2^

In the UK, the 2025 General Medical Council (GMC) systematic training survey of over 50,000 resident doctors (response rate of 70%) reported an overall satisfaction rate of 84%.^1^ However, one in five resident doctors are at high risk of burnout – more than double the proportion in 2019. Workforce sentiment appears consistent with these findings, although the problem of burnout may be even bigger. In a smaller, voluntary British Medical Association (BMA) survey of approximately 3,000 resident doctors in 2022, over half reported a low (or very low) desire to remain in the NHS within the next 12 months, and almost 60% reported low morale at the time.^3^

In Australia, national survey data are limited but suggest a similar picture. The 2025 Australian Medical Training Survey of 18,000 doctors (representing 36.7% of doctors in training) reports high overall satisfaction, with 83% of trainees recommending their workplace.^2^ However, 45% reported heavy workloads and around 30% reported experiencing or witnessing bullying, discrimination or harassment. A state-level survey in Queensland indicates that half of doctors meet the threshold for burnout risk, with burnout being a leading factor influencing intentions to leave practice, particularly in rural and regional settings.^4^

Wellbeing concerns are heightened by the data on suicide risk. In the UK, mortality data estimate that one doctor dies by suicide approximately every 3 weeks, with female doctors at a particularly higher risk.^5^, ^6^ In Australia, while the absence of a comprehensive national register limits precise estimates, multiple studies indicate elevated suicidal ideation and suicide risk among doctors, particularly junior and female clinicians.^7^

Importantly, early-career doctors appear particularly vulnerable in both systems. Newly qualified doctors experience higher levels of stress and burnout than their senior counterparts, reflecting the challenges of transition into clinical practice and demanding working environments.^8^

## Workforce pressures in practice

In the UK, workforce strain is highly visible. Repeated large-scale industrial action by both resident doctors and consultants reflects concerns not only with pay erosion, but a broader dissatisfaction with working conditions and career progression.^9^ Despite expansion of the UK senior medical workforce over recent decades, growth in training posts has not consistently kept pace with increasing demand. Consequent competition and delays mean that doctors are taking time out of training and exploring opportunities abroad, as well as increasing uptake of flexible working.^1^

In Australia, workforce pressures are less overtly publicised, but remain significant. Moral distress, heavy workloads and workplace cultural issues, including bullying and discrimination, are consistently reported.^2^ Workforce maldistribution is a key challenge, with shortages of clinicians in rural and regional areas, placing sustained pressure on services.^10^ Industrial action has occurred in Australia, including strikes in New South Wales in 2025,^11^ but remains less frequent than in the UK. This may reflect differences in system structure, with healthcare delivered at a state level, as well as variation in workforce conditions and union representation.

Remuneration for resident doctors differs substantially between the UK and Australia. While base salaries are broadly comparable in early postgraduate years, Australian systems provide more generous overall compensation through mandatory penalty rates, overtime multipliers and employer-funded superannuation. Consequently, total earnings. particularly beyond the foundation years, tend to be higher in Australia, with important implications for workforce mobility and retention (Table 2).

**Table 2:** Policy implications and opportunities for reform.

**1. Workforce planning must become a long-term national priority**
The most consistent lesson across international comparisons is that workforce shortages take many years to correct. Training a physician requires a decade or more of investment, and short-term workforce planning cycles are insufficient to address structural deficits. Countries that have maintained relatively stable workforce supply, including Australia, have generally implemented long-term training expansion and workforce monitoring. The UK’s NHS Long Term Workforce Plan represents an important step in this direction, but sustained implementation and investment will be essential. Workforce planning must also account for changing career patterns, including increasing rates of flexible and less-than-full-time working among younger clinicians.
**2. Retention should be valued as highly as recruitment**
Health systems often focus heavily on training additional doctors while overlooking the importance of retaining experienced clinicians. Yet the loss of trained doctors through burnout, early retirement or migration represents a substantial loss of expertise and investment. Retention strategies should therefore include improved job planning, flexible career pathways, reduction of administrative burden and access to professional development. Creating supportive workplace cultures that allow clinicians to sustain long and fulfilling careers may ultimately be one of the most effective workforce interventions available.
**3. Workforce wellbeing should be recognised as a core health system metric**
Historically, physician wellbeing has been viewed primarily as an individual responsibility. Increasingly, however, it is recognised as a marker of system performance. High levels of burnout are associated with reduced productivity, increased staff turnover and potential impacts on patient safety. Monitoring workforce wellbeing, alongside traditional metrics such as waiting times or service activity, could help health systems better identify early signs of workforce strain. Leadership at organisational and national levels will be essential to embed workforce wellbeing as a core indicator of healthcare system sustainability.

## Workforce capacity and system resources

Differences in workforce pressures may be partly explained by underlying variation in workforce capacity and system resources. According to Organisation for Economic Co-operation and Development (OECD) data, the UK has 3.4 practising doctors per 1,000 population, compared with 4.2 in Australia and an OECD average of 3.9.^12^, ^13^

Workforce capacity in both countries is supported by internationally trained doctors. International medical graduates (IMGs) comprise over one-third of practising doctors in Australia and, in 2023–2024, accounted for half of all newly registered doctors.^14^ Similarly, IMGs currently comprise approximately 36% of hospital doctors in the UK.^15^ These figures highlight the extent to which workforce capacity in both countries is shaped by global mobility rather than domestic workforce supply alone.

While both the UK and Australia spend a comparable proportion of their gross domestic product on healthcare (10.2% and 10.3%, respectively), Australia spends substantially more per capita (US $7,400 vs $5,500) and comparatively has a much larger private healthcare system.^12^, ^13^

Greater workforce density and higher investment may provide Australia with more flexibility in workload distribution and buffer system pressures, although there is less administrative and managerial resource than the UK. Indeed, levels of moral distress and poor wellbeing in both countries highlight that workforce wellbeing is not determined by resource levels alone, and depends on a wider range of organisational, structural and cultural factors.

## Shared structural drivers

Despite differences in workforce capacity and health system funding, the UK and Australia face similar underlying structural challenges. Demand (and cost) for healthcare continues to rise, driven by ageing populations, multimorbidity and increasing complexity of care. Increasing administrative burden remains a significant contributor to clinician dissatisfaction, reducing the time available for direct patient care. This has also driven a change in workforce composition, including the expansion of non-medical practitioner roles, reshaping clinical teams and professional boundaries, particularly within the UK.

In the UK, there has been an expansion of nursing roles into advanced practitioner roles in the last 30 years, taking over some of the tasks that were traditionally done by doctors. The introduction and expansion of the physician assistant (PA) role in the UK has been more challenging, with the Leng review^16^ highlighting structural issues with scope and accountability: the GMC has started to regulate physician assistants in 2025, with the evaluation and ongoing processes also beyond the scope of this review. In Australia there are different pressures, including doctors but especially specialist nurses doing more bureaucratic duties in operational and leadership responsibilities rather than directly clinically facing roles due to a lack of administrative support, especially in public sector settings.

In the UK and Australia, changing expectations among resident doctors and other healthcare professionals are changing workforce dynamics. Flexible working arrangements, career breaks and improved work–life balance are being increasingly prioritised, requiring health systems to adapt workforce structures accordingly. Perhaps most importantly, in both countries there is a growing mismatch between workforce expansion and training capacity. Expansion of medical school places without proportional growth in training positions is leading to growing bottlenecks, uncertainty and attrition of early-career doctors, with associated financial implications for health services.

Anecdotally, UK trainees frequently move hospital and regions within the country (due to nature of current training application systems), which can foster a sense of detachment from the hospitals/institutions in which they work. Comparatively, Australia resident doctors stay in one health service/region for longer (ie frequently study and then train within the same group of hospitals/state) and therefore may feel more embedded and invested in local services. As such, retention must be prioritised alongside recruitment. Expanding domestic workforce supply without addressing the underlying factors driving burnout, disengagement and attrition risks worsening instability rather than resolving shortages.

## From individual doctor resilience to system design and culture

Historically, responses to moral distress have often focused on individual resilience, including wellbeing programmes and stress management. While these initiatives can be helpful, they cannot substitute for systemic solutions. The persistence of moral distress across UK and Australian clinicians suggests that the issue lies not simply in individual capacity to cope, but in how healthcare systems are organised and led.

Structural workforce planning interventions remain essential. This includes ensuring that staffing levels reflect population need, aligning workforce expansion with training capacity, and reducing administrative burden to protect time for direct patient care. Promoting greater flexibility in career pathways and working patterns is critical to sustaining the workforce. However, these measures alone are unlikely to be sufficient without parallel attention to organisational culture.

There is increasing evidence highlighting the importance of creating more compassionate healthcare organisations.^17^ Leadership behaviours shape how clinicians experience pressure, raise concerns and function in teams under strain. Approaches such as compassionate leadership have been clearly associated with improved staff wellbeing, engagement and retention.^18^ This approach enables clinicians to feel listened to, valued and cared for, facilitating a greater degree of autonomy, motivation and sense of belonging within teams.

Finally, while associations between staff wellbeing and patient safety and quality of care are well evidenced, they are not consistently reflected in how health systems are evaluated. Workforce wellbeing should be considered a core measure of health system performance. Embedding this within performance frameworks will move beyond recognition of the problem towards meaningful accountability.

## Conclusions

Despite growing recognition of its importance, resident doctor wellbeing remains insufficiently prioritised within health systems. Evidence and experiences from both the UK and Australia demonstrate that burnout, low morale and intention to leave practice are widespread.

While the two countries differ in workforce capacity, system design and resources, both are experiencing similar underlying pressures. It is clear that these challenges are not transient, nor confined to individual systems, but reflect shared structural issues across healthcare. Addressing these challenges will require coordinated, system-level change, with cultural reform central to this response. Ultimately, protecting resident doctor wellbeing is not optional, but fundamental to ensuring the safety, quality and sustainability of healthcare systems.

## CRediT authorship contribution statement

**Ajay M. Verma:** Writing – review & editing, Writing – original draft, Conceptualization. **Andrew F. Goddard:** Writing – review & editing, Writing – original draft, Conceptualization. **Emily C. Taylor:** Writing – review & editing. **Neeraj B. Bhala:** Writing – original draft, Conceptualization.

## Funding

No funding was required for this article from NB, ET, AG or AV. This research did not receive any specific grant from funding agencies in the public, commercial, or not-for-profit sectors.

## Declaration of Competing Interest

The authors declare that they have no known competing financial interests or personal relationships that could have appeared to influence the work reported in this paper.

## References

[1] 〈https://www.gmc-uk.org/cdn/documents/national-training-survey-2025-results-report_pdf-111657596.pdf〉 Accessed 10/1/26.

[2] 〈https://www.medicaltrainingsurvey.gov.au〉 Accessed 10/1/26.

[3] 〈https://www.bma.org.uk/news-and-opinion/a-perfect-storm-of-poor-conditions-and-pay〉 Accessed 10/1/26.

[4] Queensland Health (2026). Medical Workforce Wellbeing Survey.

[5] Zimmermann C., Strohmaier S., Herkner H. (2024). Suicide rates among physicians compared with the general population: systematic review and meta analysis. BMJ.

[6] Gerada C., Sidhu A. (2024). Doctors and suicide. BMJ.

[7] Beyond Blue. National Mental Health Survey of Doctors and Medical Students. Melbourne; 2013.

[8] Krishnan A., Odejimi O., Bertram I., Chukowry P.S., Tadros G. (2022). A systematic review of interventions aiming to improve newly-qualified doctors’ wellbeing in the United Kingdom. BMC Psychol.

[9] 〈https://www.bma.org.uk/our-campaigns/resident-doctor-campaigns/pay-in-england/resident-doctors-guide-to-industrial-action-in-england〉 Accessed 10/1/26.

[10] Noya F., Carr S., Freeman K. (2021). Strategies to facilitate improved recruitment, development, and retention of the rural and remote medical workforce: a scoping review. Int J Health Policy Manag.

[11] 〈https://www.abc.net.au/news/2025-04-02/nsw-health-doctors-three-day-strike/105125372〉 Accessed 10/1/26.

[12] OECD (2025). Health at a Glance 2025 – United Kingdom Country Profile.

[13] OECD (2025). Health at a Glance 2025 – Australia Country Profile.

[14] 〈https://www.health.gov.au/ministers/the-hon-mark-butler-mp/media/one-doctor-every-hour-most-new-doctors-in-more-than-a-decade#:∼:text=5%2C431%20doctors%20from%20overseas%20registered,the%20last%20year%20before%20COVID〉. Accessed 10/1/26.

[15] 〈https://digital.nhs.uk/data-and-information/publications/statistical/nhs-workforce-statistics/june-2025〉 Accessed 10/1/26.

[16] The Leng review: an independent review into physician associate and anaesthesia associate professions - GOV.UK. Accessed 10/1/26.

[17] 〈https://www.kingsfund.org.uk/insight-and-analysis/long-reads/what-is-compassionate-leadership〉 Accessed 10/1/26.

[18] 〈https://www.gmc-uk.org/cdn/documents/caring-for-doctors-caring-for-patients_pdf-80706341.pdf〉 Accessed 10/1/26.

